# From COVID-19 to COVID-20: One Virus, Two Diseases

**DOI:** 10.1017/dmp.2020.363

**Published:** 2020-12

**Authors:** James J. James

In 2 prior editorials, we used the absurdist play, *Waiting for Godot,* as a metaphor for the often conflicting and illogical policies implemented to contain and mitigate the spread of coronavirus disease (COVID-19).^1,2^

Herein, we will (1) discuss how the COVID-19 pandemic has morphed into what is now, in many ways, a new disease (COVID-20), even though resulting from the same agent, severe acute respiratory syndrome coronavirus 2 (SARS-CoV-2); and (2) briefly address why a vaccine, even an effective one, may not be the silver bullet desperately awaited by those at greatest risk.

There are many reasons to consider COVID-19, today, as a distinct entity from what was experienced during the first wave at its peak in April. However, before detailing some of those reasons, we should acknowledge the level of exaggerated fear among a large percentage of the general public that often precludes a more rational discussion of the risks and overall impact of this pandemic. With or without a vaccine, such a discussion is necessary if we are to move forward, restore our socioeconomic equilibrium, and continue to lessen the medical impacts of this disease. Given that we are so hypersensitized to the term *COVID-19* and the continued, relentless reinforcement of the fear associated with it by the media, a first step in desensitization might well be a name change. This is not proposed simply as a superficial expedient, but as demonstrated below, there are cogent and objective reasons, both at the individual and the societal level, to consider this.

At the individual level, the impacts of this or any pandemic can best be measured by mortality, showing deaths as a percentage of either overall cases or of the affected population. With COVID-19, the extent and impact of the pandemic have been primarily measured in terms of “cases” defined by a positive laboratory test irrespective of the presence or absence of disease or injury, the necessary definitional elements used, a priori, to identify a medical case. This distinction goes beyond semantics when you consider that up to 80% of test positives may be asymptomatic and 85% of those with symptoms suffer a relatively mild illness.^3^ “Cases,” as defined by a positive PCR, is simply an inadequate and misleading measure of the overall medical impacts of this disease. Nevertheless, in spite of the significant flaws inherent in using daily reported lab positives to assess the impact and progress of COVID-19, the media not only continue to report this measure, but also they sensationalize it by presenting it as a raw number, out of context, without a denominator and unadjusted for age, the single most important demographic in determining individual medical outcomes.^4^ The unrelenting media pressure continues to nurture the high level of public fear and gives the false impression that a reported case today carries the same outcome and risk characteristics as a case reported in the first part of this year.

To quantify this observation and at the same time show that, in terms of medical impact, COVID-19 is a much different entity today as compared to that experienced in the United States during the first wave, we can compare case rates and mortality for the 2 waves. Using the data presented in Worldometers,^5^ for the first wave, the peak 7-day averages for daily cases (32 675) were on April 10 and for daily deaths (2256) were on April 21. The corresponding figures for the second wave are daily cases (66 781) on July 25 and 1178 deaths on August 4. Thus, from the first wave to the present, cases have approximately doubled, but deaths, the more valid measure of medical impact, have fallen by some 50%, representing not only a significant drop in actual deaths, but also a decrease in the “case” mortality rate by a factor of 4. From a country perspective and using the same data set, the population mortality after July 5 (the nadir on the 7-day moving average curve) decreased by 42% compared to that prior to July 5. Nonetheless, for political and commercial reasons, the media continue to stoke public fear with reports based on daily cases usually presented out of context. This is not to downplay the serious and too often fatal consequences of COVID-19; it is meant to put this pandemic in a more realistic context so that individuals can better assess their risks and the protective actions best taken for themselves, others, and their communities. To enable this, however, impact measures must be valid and consistent over time. One heartening observation that fits those criteria is that current treatment protocols and medical interventions have led to significantly improved survival among the most severe and critical cases.^6^

Looking at COVID-19 from the population level, there are even more cogent reasons to consider redefining what we are dealing with today versus that of several months ago. In March, when a pandemic was declared, the focus was on COVID-19 as an emerging infectious disease that we needed to control, better understand, and develop effective treatment protocols for. Governmental policies and public health interventions, such as lockdowns and school closures, were almost entirely focused on containment and mitigation with little acknowledgment of the severe socioeconomic damage that accompanied them. The effectiveness of these measures is a subject of ongoing debate, but what is not debatable is that, in the United States, there have been over 7 million reported “cases” with possibly up to 24 times that number of totally infected involving some 99% of US county-level jurisdictions.^5^ It is difficult to see how containment and mitigation efforts alone will effectively limit this disease except in isolated, well-controlled instances. Possibly, it is time to begin thinking of focusing our interventions and policies on treating another chronic, endemic disease, as opposed to eliminating a pandemic one, much as we have done with HIV/AIDS. This becomes even more necessary when we consider the full scope of the current public health crisis engendered by SARS-CoV-2.

As the pandemic evolved, so did our appreciation for those at greatest risk of negative outcomes, especially those risks not directly attributable to the disease itself and certainly not identifiable or measurable by a specific laboratory test. From the beginning, it was clear that the elderly were particularly susceptible to negative outcomes with 80% of deaths occurring in those over age 65. Soon it became equally clear that the presence of comorbidities was also a significant risk factor with 1 or more identified in 94% of lethal outcomes and showing, on average, 2.6 comorbid conditions or causes per death.^7^ In addition to these biologically inherent risk factors, it also became obvious that there was a host of others identified that significantly increased overall risk. These factors include occupational, racial, and other socioeconomic determinants of health significantly concentrated in vulnerable populations and in need of much more than a vaccine or therapeutic agent for mitigation. As a consequence, the pandemic at this stage can no longer be defined in terms of a distinct medical entity; it has evolved into a complex public health crisis(es) that can only be successfully addressed with an integrated, all-of-society effort.

A model for this type of approach can be found in the work of Merrill Singer and his concept of a “syndemic,” which is described as: “The syndemics model of health focuses on the biosocial complex, which consists of interacting, co-present, or sequential diseases and the social and environmental factors that promote and enhance the negative effects of disease interaction.”^8^ This concept clearly applies to COVID-19 today, which we can no longer consider a single biological disease entity but one that significantly overlaps and interacts with other disease conditions, as well as concurrent public health crises defined more by the socioeconomic determinants of health rather than by pathophysiological changes. The expansion of this model to include the unwanted side effects of our interventions can also give us a framework to better assess and measure the direct and indirect impacts of the COVID-19 pandemic. We are all familiar with individual iatrogenic effects that may result from medical interventions, but we have tended to ignore or minimize the very real population iatrogenic effects secondary to extreme public health interventions, such as lockdowns and school closures.

Whether or not we accept the above considerations as grounds for a name change for COVID-19, we should reasonably accept that we are dealing not with a pandemic but a syndemic. As we move closer to the availability and distribution of a vaccine, this distinction will be critical as, under the best of circumstances, adequate supplies will surely be limited necessitating an allocation plan that will surely be controversial.^9^ One would hope that those at greatest risk would have the highest priority, and medically there are 2 defining risk factors amenable to vaccine prevention: age and comorbidities. There is also a pressing need to address the critical socioeconomic mediated risk factors, but these require interventions beyond vaccination. An allocation program based on broad categorization that include large percentages of younger, healthier individuals should not receive blanket coverage at the expense of others at a far greater risk of a lethal outcome. The exception to this should only be based on a demonstration of significantly reduced vaccine effectiveness in a targeted high-risk group. Unfortunately, this might prove to be the case for the group at highest risk, the elderly, who, in addition, carry a disproportionate share of comorbidities. In addition that most vaccines are generally less effective for this demographic, in 18 SARS-CoV-2 vaccine trials conducted, the elderly have been directly excluded in 11 (61%) and indirectly in the remaining 7.^10^ Ironically, continuing in the tradition of the Theatre of the Absurd, we may well have developed vaccines that will be of the least benefit to those with the greatest need. Regardless of this potential shortcoming, we cannot lose sight that, even with the availability of a highly effective vaccine or treatment, a pharmaceutical solution is insufficient to relieve the current syndemic.
